# Anomalous behaviors of Wyrtki Jets in the equatorial Indian Ocean during 2013

**DOI:** 10.1038/srep29688

**Published:** 2016-07-20

**Authors:** Yongliang Duan, Lin Liu, Guoqing Han, Hongwei Liu, Weidong Yu, Guang Yang, Huiwu Wang, Haiyuan Wang, Yanliang Liu, Zahid, Hussain Waheed

**Affiliations:** 1 Center for Ocean and Climate Research, First Institute of Oceanography, State Oceanic Administration, Qingdao 266061, China; 2 Laboratory for Regional Oceanography and Numerical Modeling, Qingdao National Laboratory for Marine Science and Technology, Qingdao 266061, China; 3 Key Laboratory of Ocean Circulation and Waves, Institute of Oceanology, Chinese Academy of Sciences, Qingdao 266071, China; 4 Maldives Meteorological Service, Hulhulé 22000, Maldives

## Abstract

*In-situ* measurement of the upper ocean velocity discloses significant abnormal behaviors of two Wyrtki Jets (WJs) respectively in boreal spring and fall, over the tropical Indian Ocean in 2013. The two WJs both occurred within upper 130 m depth and persisted more than one month. The exceptional spring jet in May was unusually stronger than its counterpart in fall, which is clearly against the previous understanding. Furthermore, the fall WJ in 2013 unexpectedly peaked in December, one month later than its climatology. Data analysis and numerical experiments illustrate that the anomalous changes in the equatorial zonal wind, associated with the strong intra-seasonal oscillation events, are most likely the primary reason for such anomalous WJs activities.

In the tropical Indian Ocean, the equatorial surface currents reverse direction four times a year^1^. The strong eastward Wyrtki Jets (WJs), forced directly by the equatorial westerlies, occur during the monsoon transition periods of spring and fall^2^,^3^. The WJs modulate the zonal distribution of upper ocean mass, heat and salinity flux over the equatorial Indian Ocean^3^,^4^,^5^,^6^,^7^,^8^,^9^,^10^,^11^,^12^ and play key roles in the development of the Indian Ocean Dipole (IOD) events^13^,^14^,^15^,^16^,^17^,^18^.

Based on satellite and *in-situ* observations, previous studies of the WJs mostly focused on its dynamics at the sea surface and variations on short timescales. From October 2004, the Research Moored Array for African-Asian-Australian Analyses and Prediction (RAMA) began to provide amount of *in-situ* ocean current measurements, which revealed the detailed features and multi-timescale variability of the WJs, such as 3D structures, amplitudes, and transport fluxes^19^. Based on RAMA data, the seasonal to interannual variability of the WJs are widely analyzed. Researchers demonstrated that the interannual variations of the WJs are often associated with IOD and El Niño/Southern Oscillation (ENSO) events^8^,^20^,^21^,^22^,^23^,^24^,^25^,^26^,^27^. Compared with the seasonal to internannual time scale variation, intra-seasonal variability of WJs is still less studied.

In this paper, we use *in-situ* observations from the acoustic Doppler current profiler (ADCP) mooring to disclose the intra-seasonal variations of WJs during 2013 and the possible reasons. The rest of the paper is organized as follows: in section 2, observed results from the ADCP in the eastern Indian Ocean are given. We also show the link between currents and the wind forcing based on the data analysis and numerical experiments. In section 3, we provide a summary and discussion of the results of the study. Finally, the data sets and model experiments used in this research are introduced in section 4.

## Results

### The observed behaviors of WJs

**Figure 1:**
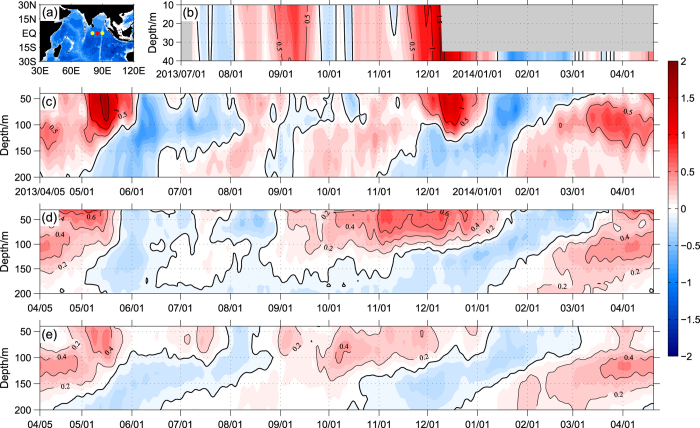
The topography of the Indian Ocean based on ETOPO5 (**a**), where the yellow (red) dots indicate the RAMA (FIO) ADCP mooring locations. Time evolution of daily zonal velocities smoothed with a 5 day running mean (shaded with interval 0.1 m s^−1^) observed by (**b**) the RAMA mooring at (0°, 80.5°E) from July 2013 to April 2014 and (**c**) FIO ADCP mooring at (0°, 85°E) from April 2013 to April 2014, with zero thick contours. Mean seasonal cycle of daily zonal velocity smoothed with a 5 day running mean (**d,e**) based on the RAMA moorings at (0°, 80.5°E) for October 2004 to August 2012 and at (0°, 90°E) for November 2000 to June 2012. Maps are generated using MATLAB R2011a (http://cn.mathworks.com/).

The zonal current evolution at 0°, 85°E observed by the ADCP mooring system is illustrated in Fig. 1c. Two strong eastward WJs occurred in the upper 130 m layer during the boreal spring and fall season of 2013. The strength of 0.5 m s^−1^ for the zonal velocity had been adopted as the criterion to indicate the jet onset and disappearance. Each of the WJs in 2013 established instantly, prevailed for more than one month, and then decayed quickly. Compared with the climatology (Fig. 1d,e), the WJs in 2013 showed some distinct characteristics. The spring WJ presented mainly in May as expected, and consistent with Iskandar *et al*.^28^ and McPhaden *et al*.^25^. The fall WJ occurred unexpectedly in the middle of November and peaked in December, which is one month later than the normal period as illustrated in previous observations (Fig. 1d,e) and described in most previous studies (e.g. ref. 22). The late arrival of the fall WJ is also captured by RAMA data. The RAMA near surface current (Fig. 1b) illustrates that the fall jet of 2013 appeared in late November and then strengthened rapidly in early December.

**Figure 2:**
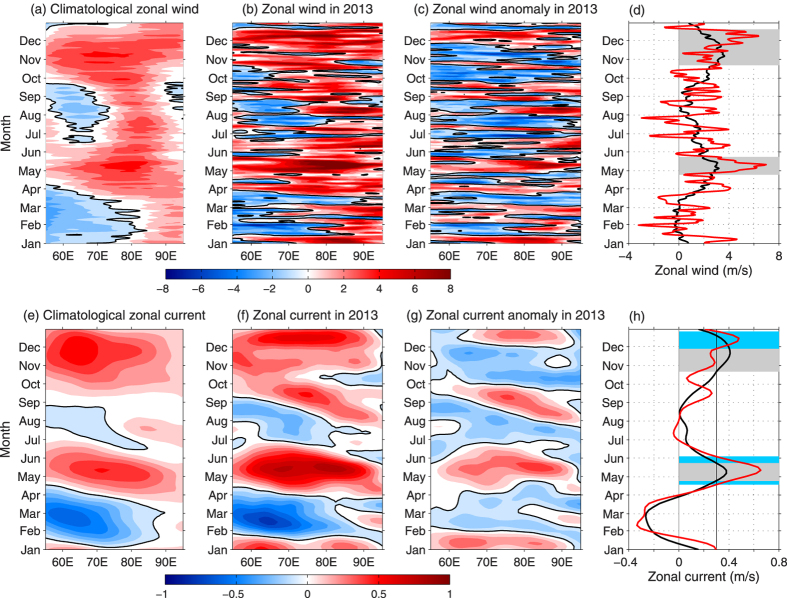
Longitude-time diagram of the climatological (**a,e**), 2013 (**b,f**) and anomalous (**c,g**) zonal surface wind (upper panel, m s^−1^) and current (bottom panel, m s^−1^) averaged over 2°S-2°N band. Time evolution of the longitudinal averaged zonal surface wind (**d**) and current (**h**). The black lines indicate the climatology, and the red lines are for year 2013 (**f**). The criterion of 0.3 m s^−1^ is adopted in (**h**) to indicate the jets occur. The semiannual eastward WJs are marked by gray bars for climatology and by light-blue bars for 2013. Maps are generated using MATLAB R2011a.

Climatologically, the fall jet is faster and more intense than that in spring as seen in both *in-situ* observations and in numerical simulations (e.g. refs 1,25 and 29, 30, 31 see also Figs 1d and 2e). Here, our observations show for the first time the evidence that the spring jet in 2013 (~1.8 m s^−1^) is much faster than that in fall (~1.4 m s^−1^), totally different from previous studies. OSCAR data analysis (not shown) and limited available ADCP *in-situ* observations^25^ also suggested that the spring jet in 2013 is more likely the strongest event than any other historical spring jets. Furthermore, it is found that the anomalous strengthened spring jet is only constrained in the ocean upper layer and the subsurface current condition remains normal. Figure 1c also shows the transient eastward equatorial undercurrents in the thermocline (50–200 m depth), which takes place in April, August-November in 2013 and March–April in 2014. The undercurrent in boreal spring (~0.7 m s^−1^) is much stronger than that in summer (~0.3 m s^−1^), which is consistent with the previous results (see also Fig. 1d,e^25^,^28^,^32^).

### Mechanism

The equatorial ocean is able to respond to westerly winds by developing an accelerating eastward jet in a few days^33^,^34^,^35^,^36^,^37^,^38^). Previous studies have widely described that the WJs in the Indian Ocean are mainly forced by the local equatorial zonal winds during the transition season between Asian summer monsoon and Asian winter monsoon^1^,^3^,^10^,^29^,^30^,^31^. For year 2013, what affects the anomalous wind along equatorial Indian Ocean? In order to check the influences of local atmospheric circulation, the anomalous equatorial surface zonal wind and ocean surface currents are shown in Fig. 2. The westerly wind in May (December) 2013 is 80% (41%) stronger than the climatological wind in May (December), and in October-November is 23% weaker than the climatological wind in October-November. Strengthened zonal winds are likely to influence the two WJs significantly (Fig. 2g–h).

**Figure 3:**
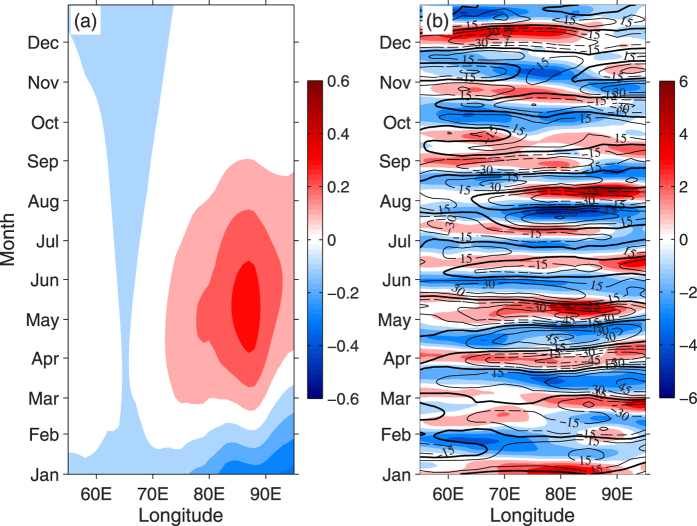
Time-longitude diagram of (**a**) interannual part (1–5 years band-pass) of zonal wind in 2013 (shaded with interval 0.1 m s^−1^), and (**b**) intraseasonal part (20–110 days band-pass) of daily zonal wind (shaded with interval 1 m s^−1^) and OLR for 2013 (contours with interval 15 W m^−2^, solid/dashed lines indicating positive/negative values), all averaged between 2°N–2°S. In this figure, different colorbars are used. Figures are plotted using MATLAB R2011a.

The fundamental feature of the WJs is linear dynamics associated with wind forcing^14^,^45^ at different timescales^27^, which reminded us to figure out whether the interannual variability of the zonal wind is responsible for the variability of the WJs. However, the interannual patterns of the zonal wind anomalies in 2013 are more uniform but much weaker (~0.3 m s^−1^) (Fig. 3a), which may suggest that the interannual part of the wind is not sufficient to induce the aforementioned anomalous behaviors of WJs in 2013.

The tropical Indian Ocean region is strongly affected by intra-seasonal oscillations (ISOs) events and the equatorial westerly anomalies are one of the significant features of ISOs^46^. ISOs are modulated by the fluctuations of the Asian summer monsoon^47^,^48^,^49^,^50^ and Asian winter monsoon. The oceanic responses to this intra-seasonal wind forcing in the Indian Ocean have been described based on numerical experiments^43^,^51^,^52^,^53^ and *in-situ* and satellite observations^36^,^37^,^38^,^44^,^54^. In order to investigate the mechanisms of the strong zonal wind anomalies in 2013, we analyzed the atmospheric data and conduced three numerical experiments to show the detailed processes associated with these wind anomalies and try to find whether ISOs in the tropical Indian Ocean is able to modulate the surface current anomalies.

As shown in Figs 3b and 2a, the amplitudes of intra-seasonal zonal wind fluctuations in 2013 were as large as the climatological components. During the WJs periods (April-May and October-December), there were several distinct positive and negative ISO events over the tropical Indian Ocean region (Fig. 3b). In particularly, the negative ISOs controlled the tropical Indian Ocean through April and November. The positive phase of ISOs prevailed from late April to mid-May and from late November to mid-December. As a result of ISO events, the surface westerly winds near the equator were particularly strong in mid-May as well as in early December, and the maximum speed is larger than 6 m/s (Figs 2d and 3b).

**Figure 4:**
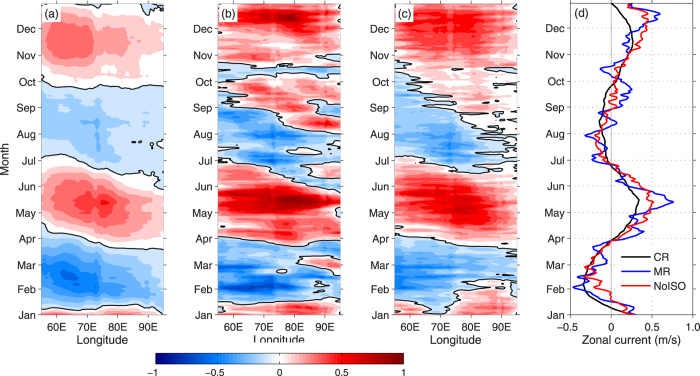
Time-longitude diagram of simulated surface zonal current (interval 0.1 m s^−1^ with zero thick contours) for CR (**a**), MR (**b**) and NoISO (**c**), all averaged ones between 2°N–2°S. Time evolution of the longitudinal averaged zonal current (**d**). The CR and MR simulations are forced by daily forcing fields from climatology and year 2013, respectively. The forcing fields in NoISO simulation are same as those in the MR except for subtracting the ISO signals. Maps are generated using MATLAB R2011a.

In the numerical experiments, the Climatology Run (CR) and Main Run (MR) well reproduce the climatological WJs and their anomalous behaviors in 2013 as observation (Fig. 4a,b). However, after removing the ISO-related wind forcing (NoISO), the spring jet in 2013 is similar as the climatology with onset at April and disappearance at June (Fig. 4c,d). The westward propagation phenomenons are both visible. Meanwhile, the fall jet begins at early November and strengthens gradually to the peak at December. Both of the WJs in NoISO are much weaker than in MR. It is suggested that the patterns of the anomalous wind associated with the ISOs play important roles on squeezing the WJ into one month (May and December) and enhancing their intensity. In conclusion, the strong ISOs appeared frequently over tropical Indian Ocean and generated significant changes in the surface westerly winds near the equator, which forced the upper ocean and the abnormal WJs occurred.

### Summary and Discussion

In this study, we present the analysis of observations for the two WJs in the eastern equatorial Indian Ocean during 2013. The results indicate there are remarkably anomalous behaviors of the WJs and the relevant atmospheric circulation in this year. Firstly, the exceptional spring jet is unexpectedly stronger than the fall jet. Secondly, the fall jet peaks in December, one month later than expected. Lastly, there are anomalous equatorial zonal winds over the tropical Indian Ocean, which may contribute to WJ changes.

The two WJs during 2013 both established rapidly, with a four- to five-fold increase in zonal velocity in only 3 days, in early May and late November. Although similar abnormal fall WJs have also been reported for late 2004 and 2011^36^,^37^,^38^, we have proposed a mechanism for the abnormal event in 2013 in this study. By conducting a series of numerical experiments, it is suggested that the zonal wind anomalies associated with the strong ISO event is able to modulate the intra-seasonal change of WJ phenomenon. The high-frequency *in-situ* current data and corresponding satellite remote observations permit us to explore further the detailed micro processes during the whole WJ period. The dynamics and effect of abnormal WJs will be described in a separate publication.

## Methods

### Measurements from the mooring system

The study utilizes data from an upward-looking 75 kHz RDI ADCP located at 0°, 85°E from 5 April 2013 to 18 April 2014 (Fig. 1c). The instrument head depths ranged between 339–405 m. Hourly averaged horizontal current velocities were recorded at 16 m vertical intervals, then gridded to 10 m resolution in upper 300 m depth. Daily averaged current data were calculated from original data. We neglect near surface measurements in the upper 35 m, which is contaminated by acoustic signals reflected at the surface layer.

The current data obtained from the RAMA moorings is also considered. Two subsurface upward-looking ADCP moorings have been deployed at 0°, 80.5°E since October 2004^19^ and at 0°, 90°E since November 2000^44^, respectively. Based on RAMA observations, the mean seasonal cycle of the zonal velocity is conducted to present the climatological characteristics of the WJs. The limited near surface current at 0°, 80.5°E measured by the current meter measurements at 10 m and 40 m depths are also derived to support our ADCP observations.

### OSCAR current data

As the ADCP observations are not able to provide the near surface current information, we adopt the 5-day averaged surface velocity data from Ocean Surface Current Analyses Real Time (OSCAR) for the surface current analysis. This data is available on a 1° × 1° grid starting from October 1992 and represents the average current at 15 m depth. This product is derived from satellite altimetry measurements of ocean surface height, surface winds, and SST, using a diagnostic model of ocean currents based on frictional and geostrophic dynamics^55^.

### Wind and OLR data

The daily surface wind data with 1° × 1° resolution from European Centre for Medium-Range Weather Forecasts Interim Reanalysis (ERA-Interim^56^) and daily interpolated outgoing longwave radiation (OLR^57^) data with 2.5° × 2.5° resolution from the National Oceanic and Atmospheric Administration (NOAA) are used to study the mechanisms associated with the changes of the WJs. The climatological annual cycle is calculated based on the available period for each data and the anomalies are obtained by subtracting the climatological annual cycles from their respective daily mean time series.

### Model setup

To assess the role of ISO-related zonal wind forcing on the WJs, three numerical experiments are also performed. The ocean general circulation model used in this study is the Princeton Ocean Model (POM), which is configured to the global ocean with a horizontal resolution of 0.5° × 0.5° and 21 vertical layers with higher resolution in the upper mixed layer. The surface forcing fields include 6 hourly surface wind and evaporation data from ERA-Interim, surface heat flux from COADS, and precipitation data from the Tropical Rainfall Measuring Mission^58^. Taking the WOA09 annual climatology of temperature and salinity as the initial condition, the model is spun up from a state of rest for 35 years using climatological forcing fields, and the mean outputs over last three year are referred to as Climatology Run (CR). Restarting from the spun-up solution, the model is integrated forward from 1 September 2012 to 31 December 2013 with the forcing fields described above. This experiment is referred to as Main Run (MR). In order to measure the ISO-related wind forcing effect in the last experiment, named as NoISO, the 20–110 day bandpass filtered signals of the wind forcing field is subtracted, and other forcing fields are same as those in the MR.

## Additional Information

**How to cite this article**: Duan, Y. *et al*. Anomalous behaviors of Wyrtki Jets in the equatorial Indian Ocean during 2013. *Sci. Rep.* **6**, 29688; doi: 10.1038/srep29688 (2016).
